# Proteomic Profiling Identifies Candidate Diagnostic Biomarkers of Hydrosalpinx in Endometrial Fluid: A Pilot Study

**DOI:** 10.3390/ijms25020968

**Published:** 2024-01-12

**Authors:** Roberto Gonzalez-Martin, Pedro de Castro, Carmen Fernandez, Fernando Quintana, Alicia Quiñonero, Marcos Ferrando, Francisco Dominguez

**Affiliations:** 1 IVIRMA Global Research Alliance, IVI Foundation, Instituto de Investigación Sanitaria La Fe (IIS La Fe), 46026 Valencia, Spain; roberto.gonzalez@ivirma.com (R.G.-M.); pedro.decastros@ivirma.com (P.d.C.); alicia.quinonero@ivirma.com (A.Q.); 2 IVIRMA Global Research Alliance, IVI-RMA Bilbao, 48940 Bizkaia, Spain; carmen.fernandez@ivirma.com (C.F.); fernando.quintana@ivirma.com (F.Q.); marcos.ferrando@ivirma.com (M.F.)

**Keywords:** hydrosalpinx, endometrial fluid, proteomic profiling, SWATH-MS, diagnostic biomarkers

## Abstract

Hydrosalpinx is a fluid occlusion and distension of the fallopian tubes, often resulting from pelvic inflammatory disease, which reduces the success of artificial reproductive technologies (ARTs) by 50%. Tubal factors account for approximately 25% of infertility cases, but their underlying molecular mechanisms and functional impact on other reproductive tissues remain poorly understood. This proteomic profiling study applied sequential window acquisition of all theoretical fragment ion spectra mass spectrometry (SWATH-MS) to study hydrosalpinx cyst fluid and pre- and post-salpingectomy endometrial fluid. Among the 967 proteins identified, we found 19 and 17 candidate biomarkers for hydrosalpinx in pre- and post-salpingectomy endometrial fluid, respectively. Salpingectomy significantly affected 76 endometrial proteins, providing insights into the enhanced immune response and inflammation present prior to intervention, and enhanced coagulation cascades and wound healing processes occurring one month after intervention. These findings confirmed that salpingectomy reverses the hydrosalpinx-related functional impairments in the endometrium and set a foundation for further biomarker validation and the development of less-invasive diagnostic strategies for hydrosalpinx.

## 1. Introduction

Tubal factors account for approximately 25% of infertility cases [1]. Hydrosalpinx is the most severe manifestation of tubal disease [1], characterized by a fluid occlusion and distension of the fallopian tubes [2]. This condition commonly affects women of reproductive age and is related to diminished pregnancy rates in patients undergoing assisted reproductive technologies (ARTs) [3]. The fallopian tubes mainly become distended due to pelvic inflammatory disease, followed by endometriosis, appendicitis, and previous pelvic or abdominal surgery. Pelvic inflammatory disease, a complication of sexually transmitted infections such as *Chlamydia trachomatis* or *Neisseria gonorrhoeae*, results in severe inflammatory processes that obstruct the distal fallopian tube [1].

The gold standard for hydrosalpinx diagnosis is salpingography. This is an invasive, expensive, and, in some cases, uninformative approach [2,3]. Therefore, the use of non-invasive techniques has been proposed to diagnose hydrosalpinx and avoid unnecessary laparoscopies. Among non-invasive techniques, ultrasound techniques (such as transvaginal ultrasound) have good specificity and sensitivity for hydrosalpinx diagnosis [3,4]. However, these techniques are sometimes unable to detect small amounts of oviductal fluid [3]. Hence, the identification of accurate noninvasive biomarkers in plasma or endometrial fluid (EF) would significantly reduce the risk, time, and cost associated with hydrosalpinx diagnosis [5].

There is rising interest in the presence and role(s) of the proteins and molecules in the EF, particularly of patients with endometriosis. Several studies sought to evaluate the degree to which these regulators affect endometriosis initiation and progression and to guide decisions regarding embryo transfer [6,7,8,9]. However, a better understanding of how hydrosalpinx affects the functions of reproductive tissues is needed. Proteomic profiling of the EF may facilitate the discovery of biomarkers for earlier, less-invasive diagnosis or prediction of ART outcomes. In clinical practice, endometrial aspirates can be obtained through minimally invasive procedures with low risk of complications. There currently exist biomarkers, such as mesothelin, to predict embryo implantation outcomes [6,7,8], but biomarkers that confirm hydrosalpinx presence and resolution remain unknown.

Hydrosalpinx cyst fluid (HCF) is embryotoxic, contains growth-factor inhibitors, and reduces pregnancy rates in patients undergoing ART [1,3]. Moreover, HCF reduces sperm motility and velocity following 24 h incubation [3,10] and thus potentially impedes fertilization in vivo. Notably, hydrosalpinx reduces ART success by 50% [11,12,13]. Andersen et al. (1994) showed that despite a comparable number of oocytes aspirated and embryos transferred following in vitro fertilization (IVF), women with hydrosalpinx had lower implantation and pregnancy rates compared to women without hydrosalpinx (2.9% vs. 10%, and 22% vs. 36%, respectively) [14]. Patients with hydrosalpinx also present higher rates of ectopic pregnancy and miscarriages [12,15]. Finally, bilateral hydrosalpinx exacerbates the risk of poor reproductive outcomes; however, the molecular mechanisms behind the worse pregnancy outcomes in hydrosalpinx females remain unknown [1].

Surgical treatment of hydrosalpinx prior to IVF and embryo transfer is indicated to minimize the detrimental effects of the HCF. The most common surgical approaches consist of ultrasound-guided HCF aspiration or complete removal of the fallopian tubes (salpingectomy). However, it remains unclear if and how salpingectomy modifies the composition of the endometrial fluid [16].

This study aims to address these gaps by characterizing the proteomic composition of the HCF and EF in patients before and after salpingectomy, determining the impact of salpingectomy on the endometrial proteome and revealing candidate diagnostic biomarkers for hydrosalpinx.

## 2. Results

### 2.1. Demographic Characteristics

The ten participants had an average age of 37.2 ± 5.2 years and BMI of 24.2 ± 5.8 kg/m^2^. Among the participants, 40% suffered a previous history of pregnancy failure, with 30% reporting at least one ectopic pregnancy. Endometriosis was prevalent in 30% of the participants, and adenomyosis in 10%. In total, 30% experienced previous abdominal cavity surgery.

### 2.2. Proteomic Profiling of Hydrosalpinx Cystic Fluid and Endometrial Fluid

A total of 967 proteins (FDR-adjusted *p*-value < 0.01) were identified via sequential window acquisition of all theoretical fragment ion spectra mass spectrometry (SWATH-MS). The complete list of these proteins, their access codes, protein names, peptides of each protein (95%), encoding gene name, reliability of the identification, and sequence coverage percentage (% Cov.), as well as the results of principal component analysis, differential analysis, and pairwise comparisons between groups, is provided in Table S1.

### 2.3. Comparison of Protein Abundance between Hydrosalpinx Cyst Fluid, Pre- and Post-Salpingectomy Endometrial Fluid

**Figure 1:**
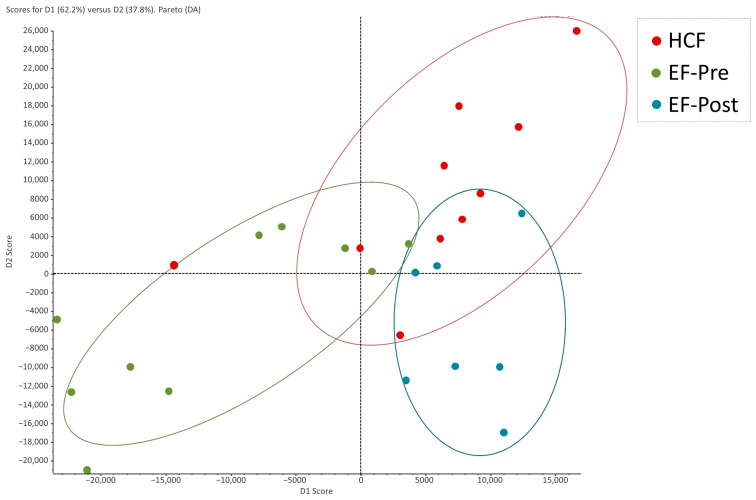
Discriminant analysis of human hydrosalpinx cyst fluid and pre- and post-salpingectomy endometrial fluid. Hydrosalpinx cyst fluid samples are represented as red dots, while pre- and post-salpingectomy endometrial fluid samples are represented as green and blue dots, respectively. HCF, hydrosalpinx cyst fluid; EF, endometrial fluid.

An exploratory discriminant analysis was carried out to determine the proteomic patterns of the samples. The proteomic patterns of pre- and post-salpingectomy EF samples were clearly distinguished by two separated clusters, while the HCF samples showed a heterogeneous pattern overlapping with the EF groups (Figure 1).

Pairwise comparisons indicated that there were 23 proteins whose abundance was significantly different in HCF and pre-salpingectomy EF (FDR-adjusted *p*-value ≤ 0.05). Of these, four proteins were overabundant in the HCF, and 19 proteins were overabundant in the pre-salpingectomy EF (Table 1 and Table S2), providing insights into the impact of hydrosalpinx on endometrial processes.

Among the four most abundant proteins in HCF, we highlight inter-alpha-trypsin inhibitor heavy chain H2 (ITIH2) (FC = 1.40, *p*-value = 0.040) and complement C5 (CO5) (FC = 1.27, *p*-value = 0.042) (Table 1 and Table S2). Of the 19 most abundant proteins in pre-salpingectomy EF, we highlight inositol-3-phosphate synthase 1 (INO1) (FC = −5.00, *p*-value = 0.038), transgelin (TAGL) (FC = −3.13, *p*-value = 0.01), and protein Niban 2 (NIBA2) (FC = −3.03, *p*-value = 0.011) (Table 1 and Table S2).

Functional enrichment analysis revealed that the most abundant proteins in the pre-salpingectomy EF were related to biological processes involved in nitric oxide biosynthesis and metabolism (Table S3).

Subsequently, we compared the protein abundance in HCF and post-salpingectomy EF to assess whether salpingectomy has the potential to reverse hydrosalpinx-related endometrial impairments. We identified 20 proteins with a significant fold change in abundance (FDR-adjusted *p*-value ≤ 0.05). Among these, three proteins were overabundant in the HCF, and 17 proteins were overabundant in the post-salpingectomy EF (Table 2 and Table S4).

We emphasize secretoglobin (SG1D2) (FC = 1.98, *p*-value = 0.023) among the three most abundant proteins in HCF (Table 2 and Table S4). From the 17 proteins most abundant in post-salpingectomy EF, we emphasized the transgelin (TAGL) (FC = −4.00, *p*-value = 0.045) and the synaptic vesicle membrane protein VAT-1 homolog (VAT1) (FC = −2.22, *p*-value = 0.026) (Table 2 and Table S4).

Functional enrichment analysis found no biological processes associated with the differentially expressed proteins.

Finally, the impact of salpingectomy on the EF composition was evaluated through the difference in protein abundance between pre- and post-salpingectomy EF samples. This comparison showed 76 proteins with significant fold change in abundance (FDR-adjusted *p*-value ≤ 0.05). Of these, 40 were overabundant in the pre-salpingectomy EF, while 36 were overabundant in the post-salpingectomy EF (Table 3 and Table S5).

Among the 40 most abundant proteins in pre-salpingectomy EF, we emphasize isoaspartyl peptidase/L-asparaginase (ASGL1) (FC = 14.57, *p*-value = 0.021), mesothelin (MSLN) (FC = 12.22, *p*-value = 0.044), and cysteine-rich secretory protein 3 (CRIS3) (FC = 10.29, *p*-value = 0.017) (Table 3 and Table S5). Within the 36 most abundant proteins in post-salpingectomy EF, we highlight tubulin beta-3 chain (TBB3) (FC = −6.67, *p*-value = 0.043), peroxiredoxin-like 2A (PXL2A) (FC = −4.17, *p*-value = 0.041), and gamma, beta, and alpha fibrinogen chains (FIGG, FIGB, FIGA) (FC = −3.13, *p*-value = 0.043; FC = −2.70, *p*-value = 0.038; FC = −2.70, *p*-value = 0.007; respectively) (Table 3 and Table S5).

**Figure 2:**
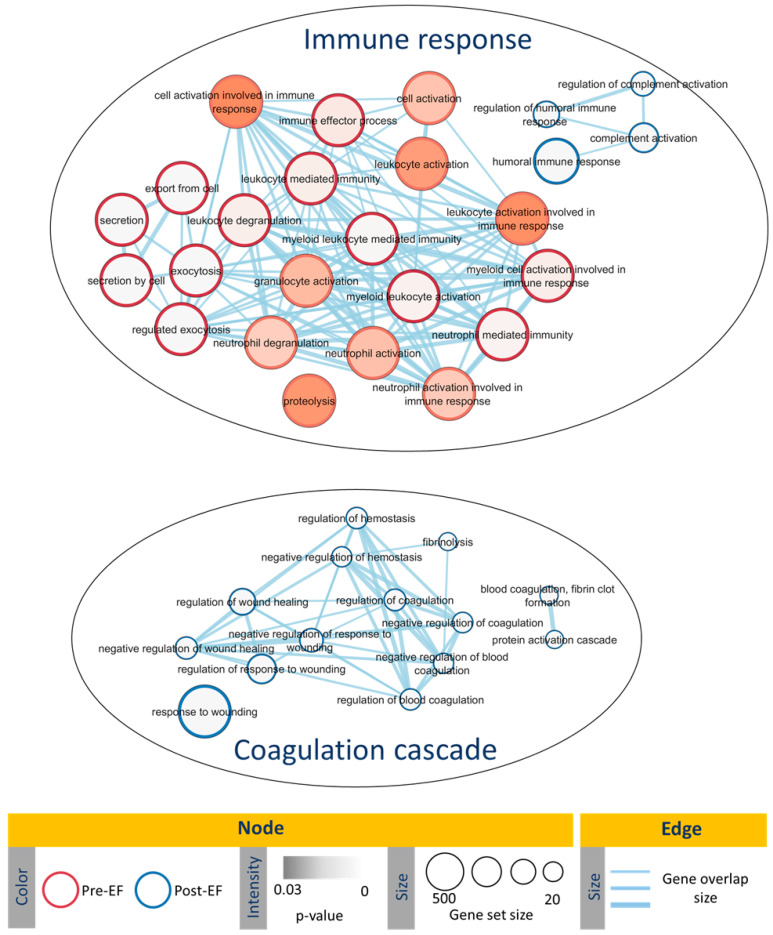
Enrichment map illustrating the significant gene ontology (GO) biological processes observed in pre- and post-salpingectomy endometrial fluid (EF). Node color represents the overexpressed group (red for pre-salpingectomy EF; blue for post-salpingectomy EF), node color intensity represents the *p*-value, and node size represents the gene set size. The edges thickness represents the gene overlap.

Functional enrichment analysis revealed that pre-salpingectomy, the most abundant EF proteins were involved in biological processes related to immune response and inflammation. Post-salpingectomy, the most abundant EF proteins were related to humoral immune response and wound healing processes (Figure 2, Table S6).

## 3. Discussion

Hydrosalpinx cyst fluid impedes early embryo development and leads to poor reproductive outcomes [1,3]. Discovering proteomic biomarkers of hydrosalpinx in the EF can lead to earlier and less invasive diagnostic strategies. Herein, we analyzed the proteomic landscape of the EF prior to and following salpingectomy to discover protein biomarkers of hydrosalpinx presence and resolution.

To determine whether the pre-salpingectomy EF was a reliable indicator of hydrosalpinx presence, we assessed the similarity of HCF and EF protein profiles. Among the four overabundant proteins identified in the HCF, we highlight inter-alpha-trypsin inhibitor heavy chain H2 (ITIH2) and other members of the ITI family which have been associated with trauma-induced inflammatory responses and breast cancer [17]. The overabundance of complement protein 5 (CO5) in the HCF is also noteworthy. Complement proteins are known to have functions beyond homeostasis and immune surveillance [18], including tissue repair and elimination of cellular debris and apoptotic cells, among other processes [19]. However, complement proteins are proinflammatory and can induce necrosis [20]. To date, there is no consensus regarding the beneficial or negative implications of overabundant complement proteins in hydrosalpinx [2]. Functional enrichment analysis showed significantly abundant proteins in the EF enriched biological processes related to the biosynthesis and metabolism of nitric oxide (NO). Interestingly, NO is produced by macrophages present in ectopic lesions and could generate a sustained inflammatory response that can promote hydrosalpinx formation [21,22].

Subsequently, we compared the proteomic profiles of HCF and post-salpingectomy EF to elucidate how fallopian tube and endometrial microenvironments are restored following salpingectomy. We distinguish SG1D2 among the three proteins with a significantly higher fold change in protein abundance in the HCF compared to the post-salpingectomy EF. This protein belongs to the Secretoglobin family, and its abundance is related to cell proliferation in carcinogenesis processes [23,24]. Thus, in this context, the overabundance of SG1D2 may be indicative of fallopian tube repair and regeneration following salpingectomy, but further studies would be required to confirm this association.

Finally, we compared the EF proteome prior to and after salpingectomy to elucidate how salpingectomy interventions alter the proteomic landscape of the endometrium and identify diagnostic protein biomarkers of hydrosalpinx. We identified 76 EF proteins that were significantly altered by salpingectomy (FDR-adjusted *p*-value < 0.05). Mesothelin (MSLN) and cysteine-rich secretory protein 3 (CRIS3) were differentially abundant prior to salpingectomy (FC = 12.22 (*p*-value = 0.044) and FC = 10.29 (*p*-value = 0.017), respectively). Notably, MSLN was previously related to epithelial malignancies and ovarian cancer [25,26]. We found a significant overabundance of both MSLN and its binding partner, MUC1, in pre-salpingectomy EF (FC = 2.43, *p*-value = 0.038), supporting a recent study proposing MSLN as a biomarker of hydrosalpinx [2]. On the other hand, CRIS3, a member of the CRISP family, is present in human exocrine secretions and secretory granules of neutrophils, and has known roles in reproductive processes [27]. Recent studies in patients with mammary carcinoma relate a lower abundance of CRIS3 to a significant improvement in disease-free survival and overall survival [28]. In this study, the 10-fold overabundance of CRIS3 in the EF prior to salpingectomy (*p*-value = 0.017) may reflect an influx of neutrophils in the endometrium, which has been shown to compromise the endometrial stroma [29,30]. Finally, when we observe the functional enrichment of these proteins, we can see that in the pre-surgery fluid, biological processes related to the immune response and inflammation appear. In the post-surgery fluid, the nature of the enriched pathways changes to pathways related to healing processes such as humoral immune response and wound healing processes.

Although our work provides new insights into the proteomic landscape of HCF and pre- and post-salpingectomy EF, it is necessary to address some limitations. First, in this pilot study, we have evaluated samples from a limited number of participants and we have not had access to a larger external validation cohort. For this reason, these results cannot be extended to the general population. Additionally, the inclusion of EF samples from a hydrosalpinx-free group would have complemented our results. However, since the main study aim was to evaluate salpingectomy’s impact on EF proteomic composition, we did not include this group in the study design. Finally, experimental validation was not feasible due to the insufficient amount of sample remaining after LC-MS/MS.

Many differences in protein profile appeared in EF before and after salpingectomy, suggesting EF as a suitable biofluid for noninvasive hydrosalpinx screening. After confirmation of our results in a larger cohort (including different degrees of hydrosalpinx and hydrosalpinx-free cases), we propose MSLN, SG1D2, and CRIS3 assessment in EF as a new first-line noninvasive screening method to assist the identification of women with hydrosalpinx, avoiding expensive invasive techniques.

## 4. Materials and Methods

### 4.1. Ethical Approval and Study Design

In this exploratory pilot study, HCF and EF samples were collected from 10 women with hydrosalpinx, diagnosed according to standard clinical protocol, at the time of salpingectomy. In addition, endometrial fluid samples were collected from seven of these women one month after surgery. Samples were not obtained from three participants because they failed to attend the post-surgical examination visit.

Participants with previous cesarean section or endometrial pathology such as uterine fibroids, polyps, asherman’s syndrome, known endometritis and/or müllerian malformations were excluded.

Sample size was established based on published studies with a similar design [2], and resources available to conduct the preliminary classification of study groups and then analyze all the samples by LC-MS/MS.

This study was approved by the Ethical Committee of IVI Bilbao (#1809-BIO-058-MF). Written informed consent was obtained from all patients.

### 4.2. Hydrosalpinx Cyst Fluid and Endometrial Fluid Collection and Processing

The hydrosalpinx cyst fluid was obtained directly from the removed surgical specimen by puncturing the dilated area of the fallopian tube by aspiration of the contents with a syringe (approx. 200 μL) once out of the patient.

Approximately 300 μL of endometrial fluid was aspirated from the endometrial cavity via a cannula inserted through the cervix immediately prior to salpingectomy (pre-salpingectomy EF; n = 10) and at a follow-up examination one month after the intervention (post-salpingectomy; n = 7 because three patients did not return for their follow-up). Samples were stored at −80 °C until they were sent to the proteomics service.

Once in the proteomics facility of the SCSIE at the University of Valencia, the HCF and EF samples were dried in a speed vacuum, resuspended in 100 μL of Laemmli sample buffer (BioRad, Madrid, Spain), and vortexed for 5 min. Samples were then sonicated for 5 min to lyse cells, heated at 95 °C for 5 min to denature proteins, and vortexed again for 5 min prior to centrifugation at 15,000 rpm and 10 °C for 20 min. The resulting supernatant of each sample was transferred to a sterile Eppendorf tube, and proteins were quantified using a quantification assay by Macherey-Nagel (Cultek, Madrid, Spain).

### 4.3. Spectral Library Construction for LC-MS/MS

To maximize the number of identified proteins, all HCF and pre- and post-salpingectomy EF samples were pooled (2 μg of each) to build a spectral library containing a total of 50 μg of protein extracts for proteomic characterization. Pooled protein extracts were loaded onto a one-dimensional SDS-polyacrylamide gel and separated by electrophoresis to determine the protein profiles of each condition. The gel was digested to construct the library for label-free quantification analysis of the study samples using SWATH-MS.

#### 4.3.1. In-Gel Protein Digestion

The gel lane with the pooled samples was divided into five fragments. Each gel fraction was digested with sequencing grade trypsin (Promega, Madrid, Spain) as previously described [31]. Briefly, each sample was digested with 500 ng of trypsin in an overnight incubation at 37 °C. The digestion was stopped with trifluoroacetic acid (TFA; at a final concentration of 10%). After removing the supernatant, the library gel slices were dehydrated with pure acetonitrile (ACN). The new peptide solutions were combined with the corresponding supernatant. All peptide solutions were dried using a rotary evaporator and resuspended with 20 μL of 2% ACN and 0.1% TFA for LC-MS/MS.

#### 4.3.2. LC-MS/MS

Five microliters of each peptide mixture was loaded onto a trap NanoLC column (3 μm particle size C18-CL, 350 μm × 0.5 mm; Eksigent, Dublín, CA, USA), purified, and concentrated with 0.1% TFA at 5 μL/min for 5 min. Next, peptides were eluted onto an analytical column (LC Column, 3 μm particle size C18-CL, 120 Å, 75 μm × 15 cm, Nikkyo, Tokyo, Japan) and equilibrated in 5% ACN and 0.1% formic acid (FA) with a linear gradient of 7% to 40% B in A over 45 min (A: 0.1% FA; B: ACN, 0.1% FA) at a flow rate of 300 nL/min. Eluted peptides were analyzed using a nanoESI qQTOF mass spectrometer (TripleTOF^®^ 6600+, AB SCIEX, Madrid, Spain).

For LC-MS/MS, samples were ionized in a Source Type Optiflow <1 μL Nano, applying 3.0 kV to the spray emitter at 200 °C. The analysis was conducted in a data-dependent mode, with survey MS1 scans acquired from 350–1400 *m*/*z* for 250 ms. The quadrupole resolution was set to ‘LOW’ for MS2 experiments, which were acquired from 100–1500 *m*/*z* for 25 ms in ‘high sensitivity’ mode. The switch criteria included a charge 2+ to 4+, minimum intensity, and 250 counts per second (cps). Up to 100 ions were selected for fragmentation after each survey scan. Dynamic exclusion was set to 15 s. To align with the CE values used in subsequent SWATH-MS experiments, the rolling collision energies (CE) equations were set for all ions as for 2+ ions according to the following equation: |CE| = (slope) × (*m*/*z*) + (intercept). A calibration curve was built using PepCalMix (2 × 10^−15^ mol) spiked with 500 ng of trypsin-digested k562 cell lysate (AB SCIEX, Spain, Madrid) to test the system’s sensitivity of acquisition.

#### 4.3.3. Peptide Query Parameters and Peptide-Centric Scoring

The .wiff files generated on the TripleTOF^®^ 6600 were processed using default parameters of the search engine ProteinPilot v5.0. (AB SCIEX, Madrid, Spain) to generate a peak list. The Paragon algorithm (4.0.0.0, 4767, [32]) of ProteinPilot v5.0. was used to search the SWISS-PROT protein database, considering the trypsin specificity, iodine acetamide cys-alkylation, and *Homo sapiens* taxonomy restriction. Data were corrected for multiple testing using false discovery rate (FDR) estimation.

To avoid using the same spectral evidence in more than one protein, the identified proteins were clustered using the Pro-Group™ algorithm. This approach pooled proteins with shared MS/MS spectra, regardless of the assigned peptide’s sequence, distinguishing proteins in high-quality peak groups. Only the group proteins for which there was individual evidence (single peptides with sufficient confidence) were listed.

### 4.4. Proteomic Profiling of Hydrosalpinx Cyst Fluid, Pre- and Post- Salpingectomy Endometrial Fluid

Once the spectral library of the proteins present in the different types of fluids was generated, the individualized identification of the quantitative profile of each type of fluid was carried out.

#### 4.4.1. In-Gel Protein Digestion and Peptide Purification of Individual Samples

For individual SWATH analysis, ten micrograms of each total protein extract was loaded onto a one-dimensional SDS-polyacrylamide gel to separate proteins by electrophoresis. Each gel fraction was separated and digested with sequencing grade trypsin (Promega, Madrid, Spain), as previously described [31]. Briefly, 500 ng of trypsin in 150 μL of Amoium BiCarbonate (ABC) solution was used to digest each sample. The enzymatic digestion was stopped with TFA (final concentration of 1%). Following a double extraction with ACN, all peptide solutions were purified using a rotary evaporator. Finally, the samples were resuspended with 20 μL of 2% ACN and 0.1% TFA in preparation for LC-MS/MS.

#### 4.4.2. SWATH-MS

The protein profiles of HCF and pre- and post-salpingectomy EF were performed by SWATH-MS, a variant of data-independent acquisition mass spectrometry. In this method, all ions within a selected *m*/*z* range are fragmented together and analyzed in a second stage of MS/MS [33,34].

Five microliters of each sample were loaded onto a trap column (NanoLC column, 3 μm C18-CL, 120 Å, 350 μm × 0.5 mm, Eksigent, Dublín, CA, USA), purified, and desalted with 0.1% TFA at 5 μL/min for 5 min. The purified peptides were loaded onto an analytical column (LC column, 3 μm C18-CL, 120 Å, 0.075 × 150 mm, Eksigent, Dublín, CA, USA) equilibrated in 5% ACN 0.1% FA. Peptides were eluted with a linear gradient of 7% to 40% B in A over 45 min (A: 0.1% FA, B: ACN, 0.1% FA) at a flow rate of 300 nL/min. Eluted peptides were injected in the nanoESI qQTOF mass spectrometer (TripleTOF^®^ 6600+, AB SCIEX, Madrid, Spain) and analyzed in SWATH mode.

Samples were ionized in a Source Type Optiflow <1 μL Nano, applying 3.0 kV to the spray emitter at 200 °C. The tripleTOF was operated in swath mode, in which a 0.050 s TOF MS scan from 350–1250 *m*/*z* was performed, followed by 0.080 s product ion scans from 350–1250 *m*/*z*. A total of 100 variable windows from 400 to 1250 *m*/*z* were acquired throughout the experiment. The total cycle time was 2.79 secs. The individual SWATH injections were randomized.

#### 4.4.3. SWATH-MS Data Analysis

SWATH-MS data were analyzed using PeakView^®^ software (v2.2, AB SCIEX, Madrid, Spain). Only peptides annotated with at least 95% confidence and an FDR-adjusted *p*-value ≤ 0.01 were selected for analysis. The maximum number of analyzed peptides is set to 20 for each protein. For every peptide meeting these conditions, the chromatographic area of 6 transitions (or MS/MS fragments) was integrated. The chromatographic area of the transitions was then converted into a value for the corresponding peptide, and with the peptide areas, the total protein area was estimated. Retention times of the detected peptides were alienated using major proteins identified.

### 4.5. Statistical Analyses

Protein expression trends were visualized and analyzed with MarkerView (SCIEX, Framingham, MA, USA). Estimated protein areas were normalized by the sum of all quantified proteins areas. Principal component analysis and discriminant analysis, both with Pareto scaling, were applied to reduce dimensionality of proteomic profiles. Finally, differential expression of protein abundance was evaluated using *t*-tests followed by pair-wise comparisons. In all cases, FDR-adjusted *p*-values < 0.05 were considered statistically significant.

### 4.6. Functional Enrichment Analysis

Biological processes that were significantly enriched by the differentially expressed proteins in HCF and pre- and post-salpingectomy EF were identified with g:Profiler (https://biit.cs.ut.ee/gprofiler/gost (accessed on 10 Jun 2021)) using default parameters and selecting *Homo sapiens* as a reference organism. Identified biological processes were visualized with the Cytoscape EnrichmentMap plugin [35].

## 5. Conclusions

In summary, this proteomic profiling study identified differentially expressed proteins and their related biological functions in the HCF and EF of patients with hydrosalpinx prior to and after salpingectomy. We propose candidate protein biomarkers in the EF, particularly MSTL and CRIS3, for a less-invasive diagnosis of hydrosalpinx in reproductive-aged women. Finally, this study confirmed that salpingectomy reverses hydrosalpinx-related functional impairments to reproductive processes.

## Figures and Tables

**Table 1:** Top differentially abundant proteins with significant fold change in abundance between hydrosalpinx cyst fluid and pre-salpingectomy endometrial fluid.

Accession Code	Protein Name	Protein Description	*p*-Value	FC (HCF vs. Pre-Salpingectomy EF)
P18206	VINC	Vinculin	0.037	1.51
P0DOX3	IGD	Immunoglobulin delta heavy chain	0.016	1.48
P19823	ITIH2	Inter-alpha-trypsin inhibitor heavy chain H2	0.040	1.40
P01031	CO5	Complement C5	0.042	1.27
Q9NRX4	PHP14	14 kDa phosphohistidine phosphatase	0.038	−2.27
P0DMV9	HS71B	Heat shock 70 kDa protein 1B	0.014	−2.33
Q08211	DHX9	ATP-dependent RNA helicase A	0.024	−2.33
P00966	ASSY	Argininosuccinate synthase	0.038	−2.63
O14773	TPP1	Tripeptidyl-peptidase 1	0.020	−2.63
O94760	DDAH1	N(G),N(G)-dimethylarginine dimethylaminohydrolase 1	0.040	−3.03
Q96TA1	NIBA2	Protein Niban 2	0.011	−3.03
Q01995	TAGL	Transgelin	0.010	−3.13
P68371	TBB4B	Tubulin beta-4B chain	0.047	−3.85
Q9NPH2	INO1	Inositol-3-phosphate synthase 1	0.038	−5.00

The table lists the accession codes obtained from the SWISS-PROT protein database, protein name and description, *p*-values from pair-wise comparison, and the fold change (FC) of top protein abundance in pre-salpingectomy endometrial fluid (EF) with respect to hydrosalpinx cyst fluid (HCF). See Table S2 for the whole list of differentially abundant proteins.

**Table 2:** Top differentially abundant proteins with significant fold change in abundance between hydrosalpinx cyst fluid and post-salpingectomy endometrial fluid.

Accession Code	Protein Name	Protein Description	*p*-Value	FC (HCF vs. Post-Salpingectomy EF)
O95969	SG1D2	Secretoglobin family 1D member 2	0.023	1.98
P60891	PRPS1	Ribose-phosphate pyrophosphokinase 1	0.043	1.71
Q92530	PSMF1	Proteasome inhibitor PI31 subunit	0.040	1.38
Q96G03	PGM2	Phosphoglucomutase-2	0.031	−1.61
P15169	CBPN	Carboxypeptidase N catalytic chain	0.025	−1.79
P30085	KCY	UMP-CMP kinase	0.037	−2.00
Q08211	DHX9	ATP-dependent RNA helicase A	0.038	−2.08
Q86Z20	CC125	Coiled-coil domain-containing protein 125	0.040	−2.08
Q99536	VAT1	Synaptic vesicle membrane protein VAT-1 homolog	0.026	−2.22
Q15582	BGH3	Transforming growth factor-beta-induced protein ig-h3	0.050	−2.38
Q15113	PCOC1	Procollagen C-endopeptidase enhancer 1	0.032	−2.63
O14773	TPP1	Tripeptidyl-peptidase 1	0.042	−2.70
Q01995	TAGL	Transgelin	0.045	−4.00

The table lists the accession codes obtained from the SWISS-PROT protein database, protein name and description, *p*-values from pair-wise comparison, and the fold change (FC) of top protein abundance in post-salpingectomy endometrial fluid (EF) with respect to hydrosalpinx cyst fluid (HCF). See Table S4 for the whole list of differentially abundant proteins.

**Table 3:** Top differentially abundant endometrial fluid proteins with significant fold change in abundance following salpingectomy.

Accession Code	Protein Name	Protein Description	*p*-Value	FC (Pre- vs. Post-Salpingectomy EF)
Q7L266	ASGL1	Isoaspartyl peptidase/L-asparaginase	0.021	14.57
Q13421	MSLN	Mesothelin	0.044	12.22
P54108	CRIS3	Cysteine-rich secretory protein 3	0.017	10.29
P10909	CLUS	Clusterin	0.013	9.70
Q53GD3	CTL4	Choline transporter-like protein 4	0.010	6.57
Q08380	LG3BP	Galectin-3-binding protein	0.034	6.42
Q9NPH2	INO1	Inositol-3-phosphate synthase 1	0.031	5.83
Q13938	CAYP1	Calcyphosin	0.037	5.71
Q9BW30	TPPP3	Tubulin polymerization-promoting protein family member 3	0.045	5.62
P08294	SODE	Extracelular superoxide dismutase [Cu-Zn]	0.033	5.18
P67936	TPM4	Tropomyosin alpha-4 chain	0.033	−2.33
P02647	APOA1	Apolipoprotein A-I	0.001	−2.63
P02671	FIBA	Fibrinogen alpha chain	0.007	−2.70
P02675	FIBB	Fibrinogen beta chain	0.038	−2.70
P00738	HPT	Haptoglobin	0.046	−3.03
P02679	FIBG	Fibrinogen gamma chain	0.043	−3.13
P02538	K2C6A	Keratin, type II cytoskeletal 6A	0.043	−3.70
Q9BRX8	PXL2A	Peroxiredoxin-like 2A	0.041	−4.17
P08572	CO4A2	Collagen alpha-2(IV) chain	0.049	−4.17
Q13509	TBB3	Tubulin beta-3 chain	0.043	−6.67

The table lists the accession codes obtained from the SWISS-PROT protein database, protein name and description, *p*-values from pair-wise comparison, and the fold change (FC) of top protein abundance in pre-salpingectomy endometrial fluid (EF) with respect to post-salpingectomy endometrial fluid (EF). See Table S5 for the whole list of differentially abundant proteins.

## Data Availability

The data presented in this study are openly available in Mendeley Data at [doi—10.17632/ky483zmk8p.1].

## Supplementary Materials

The following supporting information can be downloaded at https://www.mdpi.com/article/10.3390/ijms25020968/s1.
