# Supplementary material for: Proteomic Profiling Identifies Candidate Diagnostic Biomarkers of Hydrosalpinx in Endometrial Fluid: A Pilot Study
Source: Int J Mol Sci. 2024 Jan 12;25(2):968. doi: 10.3390/ijms25020968 (PMC10816103; doi:10.3390/ijms25020968)
Supplement: Supplementary file 1 [file ijms-25-00968-s001.zip › Table S2.pdf]

Table S2. Proteins with significant fold change in abundance between hydrosalpinx cyst fluid and pre-salpingectomy endometrial fluid.

Accesion Code	Protein Name	Protein description	p-value	FC (HCF vs. pre-salpingectomy EF)
P18206	VINC	Vinculin	0.037	1.51
P0DOX3	IGD	Immunoglobulin delta heavy chain	0.016	1.48
P19823	ITIH2	Inter-alpha-trypsin inhibitor heavy chain H2	0.040	1.40
P01031	CO5	Complement C5	0.042	1.27
P63000	RAC1	Ras-related C3 botulinum toxin substrate 1	0.035	-1.54
P28066	PSA5	Proteasome subunit alpha type-5	0.046	-1.54
P22234	PUR6	Multifunctional protein ADE2	0.035	-1.56
P04632	CPNS1	Calpain small subunit 1	0.032	-1.69
Q96G03	PGM2	Phosphoglucomutase-2	0.014	-1.89
P62879	GBB2	Guanine nucleotide-binding protein G(I)/G(S)/G(T) subunit beta-2	0.042	-2.04
P23381	SYWC	Tryptophan--tRNA ligase, cytoplasmic	0.008	-2.04
Q07960	RHG01	Rho GTPase-activating protein 1	0.041	-2.08
P48637	GSHB	Glutathione synthetase	0.015	-2.17
Q9NRX4	PHP14	14 kDa phosphohistidine phosphatase	0.038	-2.27
P0DMV9	HS71B	Heat shock 70 kDa protein 1B	0.014	-2.33
Q08211	DHX9	ATP-dependent RNA helicase A	0.024	-2.33
P00966	ASSY	Argininosuccinate synthase	0.038	-2.63
O14773	TPP1	Tripeptidyl-peptidase 1	0.020	-2.63
O94760	DDAH1	N(G),N(G)-dimethylarginine dimethylaminohydrolase 1	0.040	-3.03
Q96TA1	NIBA2	Protein Niban 2	0.011	-3.03
Q01995	TAGL	Transgelin	0.010	-3.13
P68371	TBB4B	Tubulin beta-4B chain	0.047	-3.85
Q9NPH2	INO1	Inositol-3-phosphate synthase 1	0.038	-5.00

The table lists the accession codes obtained from Swissprot protein database protein name and description, p-values from pair-wise comparison, and the fold change (FC) of protein abundance in pre-salpingectomy endometrial fluid (EF) with respect to hydrosalpinx cyst fluid (HCF).
