# Supplementary material for: Proteomic Profiling Identifies Candidate Diagnostic Biomarkers of Hydrosalpinx in Endometrial Fluid: A Pilot Study
Source: Int J Mol Sci. 2024 Jan 12;25(2):968. doi: 10.3390/ijms25020968 (PMC10816103; doi:10.3390/ijms25020968)
Supplement: Supplementary file 1 [file ijms-25-00968-s001.zip › Table S4.pdf]

Table S4. Proteins with significant fold change in abundance between hydrosalpinx cyst fluid and post-salpingectomy endometrial fluid.

Accesion Code	Protein Name	Protein description	p-value	FC (HCF vs. post-salpingectomy EF)
O95969	SG1D2	Secretoglobin family 1D member 2	0.023	1.98
P60891	PRPS1	Ribose-phosphate pyrophosphokinase 1	0.043	1.71
Q92530	PSMF1	Proteasome inhibitor PI31 subunit	0.040	1.38
P27169	PON1	Serum paraoxonase/arylesterase 1	0.025	-1.33
P19823	ITIH2	Inter-alpha-trypsin inhibitor heavy chain H2	0.014	-1.37
P00734	THRB	Prothrombin	0.010	-1.43
P35858	ALS	Insulin-like growth factor-binding protein complex acid labile subunit	0.007	-1.56
P05090	APOD	Apolipoprotein D	0.004	-1.56
P00747	PLMN	Plasminogen	0.019	-1.59
P08236	BGLR	Beta-glucuronidase	0.024	-1.61
Q96G03	PGM2	Phosphoglucomutase-2	0.031	-1.61
P15169	CBPN	Carboxypeptidase N catalytic chain	0.025	-1.79
P30085	KCY	UMP-CMP kinase	0.037	-2.00
Q08211	DHX9	ATP-dependent RNA helicase A	0.038	-2.08
Q86Z20	CC125	Coiled-coil domain-containing protein 125	0.040	-2.08
Q99536	VAT1	Synaptic vesicle membrane protein VAT-1 homolog	0.026	-2.22
Q15582	BGH3	Transforming growth factor-beta-induced protein ig-h3	0.050	-2.38
Q15113	PCOC1	Procollagen C-endopeptidase enhancer 1	0.032	-2.63
O14773	TPP1	Tripeptidyl-peptidase 1	0.042	-2.70
Q01995	TAGL	Transgelin	0.045	-4.00

The table lists the accession codes obtained from Swissprot protein database protein name and description, p-values from pair-wise comparison, and the fold change (FC) of protein abundance in post-salpingectomy endometrial fluid (EF) with respect to hydrosalpinx cyst fluid (HCF).
