# Supplementary material for: Proteomic Profiling Identifies Candidate Diagnostic Biomarkers of Hydrosalpinx in Endometrial Fluid: A Pilot Study
Source: Int J Mol Sci. 2024 Jan 12;25(2):968. doi: 10.3390/ijms25020968 (PMC10816103; doi:10.3390/ijms25020968)
Supplement: Supplementary file 1 [file ijms-25-00968-s001.zip › Table S5.pdf]

Table S5. Endometrial fluid proteins with significant fold change in abundance following salpingectomy.

Accesion Code	Protein Name	Protein description	p-value	FC (pre- vs. post-salpingectomy EF)
Q7L266	ASGL1	Isoaspartyl peptidase/L-asparaginase	0.021	14.57
Q13421	MSLN	Mesothelin	0.044	12.22
P54108	CRIS3	Cysteine-rich secretory protein 3	0.017	10.29
P10909	CLUS	Clusterin	0.013	9.70
Q53GD3	CTL4	Choline transporter-like protein 4	0.010	6.57
Q08380	LG3BP	Galectin-3-binding protei	0.034	6.42
Q9NPH2	INO1	Inositol-3-phosphate synthase 1	0.031	5.83
Q13938	CAYP1	Calcyphosin	0.037	5.71
Q9BW30	TPPP3	Tubulin polymerization-promoting protein family member 3	0.045	5.62
P08294	SODE	Extracellular superoxide dismutase [Cu-Zn]	0.033	5.18
P12821	ACE	Angiotensin-converting enzyme	0.044	4.45
P41222	PTGDS	Prostaglandin-H2 D-isomerase	0.009	4.01
Q5VW32	BROX	BRO1 domain-containing protein BROX	0.015	3.67
O00560	SDCB1	Syntenin-1	0.013	3.33
Q86WI1	PKHL1	Fibrocystin-L	0.010	3.24
P20827	EFNA1	Ephrin-A1	0.042	3.14
P15311	EZRI	Ezrin	0.039	3.09
P36222	CH3L1	Chitinase-3-like protein 1	0.011	2.93
P00966	ASSY	Argininosuccinate synthase	0.032	2.83
Q16651	PRSS8	Prostasin	0.018	2.82
P48506	GSH1	Glutamate--cysteine ligase catalytic subunit	0.008	2.79
O94760	DDAH1	N(G),N(G)-dimethylarginine dimethylaminohydrolase 1	0.049	2.76
Q14393	GAS6	Growth arrest-specific protein 6	0.039	2.74
P15941	MUC1	Mucin-1	0.038	2.43
P13987	CD59	CD59 glycoprotein	0.025	2.36
P43251	BTD	Biotinidase	0.025	2.25
Q8N2S1	LTBP4	Latent-transforming growth factor beta-binding protein 4	0.034	2.25
P0DMV9	HS71B	Heat shock 70 kDa protein 1B	0.016	2.21
O60888	CUTA	Protein CutA	0.047	2.19
Q9UBQ7	GRHPR	Glyoxylate reductase/hydroxypyruvate reductase	0.049	2.13
Q9Y265	RUVB1	RuvB-like 1	0.042	2.06
P00568	KAD1	Adenylate kinase isoenzyme 1	0.027	1.97
P61916	NPC2	NPC intracellular cholesterol transporter 2	0.050	1.91
P04632	CPNS1	Calpain small subunit 1	0.020	1.83
P07225	PROS	Vitamin K-dependent protein S	0.017	1.80
P22061	PIMT	Protein-L-isoaspartate(D-aspartate) O-methyltransferase	0.050	1.80
Q10588	BST1	ADP-ribosyl cyclase/cyclic ADP-ribose hydrolase 2	0.034	1.70
P25774	CATS	Cathepsin S	0.019	1.69
Q14254	FLOT2	Flotillin-2	0.049	1.65

O43490	PROM1	Prominin-1	0.045	1.61
P02753	RET4	Retinol-binding protein 4	0.028	-1.35
P02766	TTHY	Transthyretin	0.009	-1.43
P02747	C1QC	Complement C1q subcomponent subunit C	0.048	-1.49
P05546	HEP2	Heparin cofactor 2	0.041	-1.54
P01031	CO5	Complement C5	0.011	-1.56
P02760	AMBP	Protein AMBP	0.043	-1.61
P19827	ITIH1	Inter-alpha-trypsin inhibitor heavy chain H1	0.002	-1.64
O95445	APOM	Apolipoprotein M	0.009	-1.67
Q9BXS6	NUSAP	Nucleolar and spindle-associated protein 1	0.022	-1.69
P00734	THRB	Prothrombin	0.001	-1.69
P27169	PON1	Serum paraoxonase/arylesterase 1	<0.001	-1.79
P04114	APOB	Apolipoprotein B-100	0.019	-1.79
P07357	CO8A	Complement component C8 alpha chain	0.049	-1.82
P05090	APOD	Apolipoprotein D	0.001	-1.89
P19823	ITIH2	Inter-alpha-trypsin inhibitor heavy chain H2	<0.001	-1.92
P01023	A2MG	Alpha-2-macroglobulin	0.047	-1.92
P36871	PGM1	Phosphoglucomutase-1	0.042	-2.04
P03952	KLKB1	Plasma kallikrein	0.010	-2.04
P15169	CBPN	Carboxypeptidase N catalytic chain	0.018	-2.04
P02656	APOC3	Apolipoprotein C-III	0.020	-2.08
P02745	C1QA	Complement C1q subcomponent subunit A	0.032	-2.08
P00747	PLMN	Plasminogen	0.003	-2.13
P35858	ALS	Insulin-like growth factor-binding protein complex acid labile subunit	<0.001	-2.13
P51884	LUM	Lumican	0.011	-2.17
P08236	BGLR	Beta-glucuronidase	0.002	-2.27
P67936	TPM4	Tropomyosin alpha-4 chain	0.033	-2.33
P02647	APOA1	Apolipoprotein A-I	0.001	-2.63
P02671	FIBA	Fibrinogen alpha chain	0.007	-2.70
P02675	FIBB	Fibrinogen beta chain	0.038	-2.70
P00738	HPT	Haptoglobin	0.046	-3.03
P02679	FIBG	Fibrinogen gamma chain	0.043	-3.13
P02538	K2C6A	Keratin, type II cytoskeletal 6A	0.043	-3.70
Q9BRX8	PXL2A	Peroxiredoxin-like 2A	0.041	-4.17
P08572	CO4A2	Collagen alpha-2(IV) chain	0.049	-4.17
Q13509	TBB3	Tubulin beta-3 chain	0.043	-6.67

The table lists the accession codes obtained from Swissprot protein database protein name and description, p-values from pair-wise comparison, and the fold change (FC) of protein abundance in pre-salpingectomy endometrial fluid (EF) with respect to post-salpingectomy endometrial fluid (EF).
